# Communication skills as a bridge between medical and public health education: the case of Greek medical students

**DOI:** 10.3389/fpubh.2025.1709045

**Published:** 2025-10-09

**Authors:** Stefanos Karakolias, Georgios Tagarakis, Nikolaos Polyzos

**Affiliations:** ^1^Department of Nursing, Democritus University of Thrace, Alexandroupolis, Greece; ^2^Department of Medicine, Aristotle University of Thessaloniki, Thessaloniki, Greece; ^3^Department of Medicine, Democritus University of Thrace, Alexandroupolis, Greece

**Keywords:** communication skills, provider–patient communication, medical education, public health education, medical curriculum, medical students, Greece

## Abstract

Effective communication skills are a critical yet often neglected bridge between individual-focused medical education and population-oriented public health goals. Using the Greek medical system as a case study, this paper highlights a significant gap in formal training, finding that only three of the seven medical schools offer dedicated communication modules, with just two being core courses and representing less than 1% of the total ECTS credits. The study contends that this lack of prioritization undermines patient-centered care and broader public health education. Effective provider–patient communication is shown to foster trust, improve treatment adherence, and enhance health literacy, which empowers individuals and communities to make informed health decisions. The study concludes by calling for systematic curricular reform, advocating for the integration of communication and health literacy as core, longitudinal components throughout medical education. It recommends employing simulation-based learning, continuous assessment, and faculty development to ensure that future physicians are equipped to simultaneously improve individual patient outcomes while advancing broader population health goals.

## 1 Introduction

As a social science discipline, communication investigates the processes through which information is exchanged within societies and among individuals (1, 2). The field integrates interpersonal and mass communication, encompassing not only the content of messages but also the channels through which they are conveyed. With the proliferation of digital technologies, social media platforms, and computational approaches, communication studies are undergoing rapid and substantial transformations (3, 4).

Health communication is the practice of providing individuals and communities with the knowledge, skills, and resources needed to make informed decisions about their health and wellbeing. By fostering understanding, motivation, and empowerment, it plays a pivotal role in advancing public health (5, 6) and improving individual health outcomes (7, 8). It is obvious that effective health communication relies on strong communication skills to convey information clearly and engage audiences.

Medical education trains healthcare professionals to diagnose, treat, and manage diseases in individual patients, making it inherently patient-centered and treatment-oriented. In contrast, public health education aims to prevent disease and promote health across populations, rendering it community-centered and prevention-oriented. While the two fields complement each other by linking individual care to population health outcomes (9), they remain conceptually and operationally distinct.

In this perspective, we discuss how communication skills among medical doctors can bridge the gap between medical and public health education. Using the case of Greek medical students, we aim to inform policymakers about current advances and future directions for fostering these skills early in medical education.

## 2 Effective provider–patient communication

Provider–patient communication, a key component of health communication, is fundamental to effective healthcare delivery. Prior research has consistently demonstrated that clear, empathetic, and culturally sensitive communication fosters trust between providers and patients, strengthening the therapeutic relationship and encouraging patients to engage more openly in discussions about their health. This open dialogue not only facilitates shared decision-making but also promotes adherence to treatment regimens, contributing to improved clinical outcomes (10, 11).

The importance of effective communication is particularly evident in the management of chronic conditions, such as diabetes, hypertension, and cardiovascular disease. Studies have shown that when providers communicate clearly and empathetically, patients are more likely to follow prescribed treatments, monitor their symptoms, and adopt necessary lifestyle modifications, enhancing both treatment adherence and self-management (12). Furthermore, communication that is responsive to patients’ unique needs and tailored to diverse populations can reduce health disparities by promoting equitable access to information, fostering understanding, and supporting patient engagement in health care decisions (13).

Beyond improving adherence and health outcomes, high-quality provider–patient communication is central to patient-centered care. By acknowledging and integrating patients’ beliefs, values, and preferences, providers can create a collaborative environment that strengthens decision-making processes, enhances satisfaction, and elevates the overall quality of care (14, 15). In this sense, communication is not merely a tool for information exchange but a foundational skill that underpins the therapeutic alliance, shapes health behaviors, and ultimately contributes to better population and individual health outcomes.

## 3 Communication skills among medical doctors

Communication skills are of paramount importance for medical doctors, as they are fundamental to ensuring high-quality patient care and favorable health outcomes. Among these, active listening is widely regarded as a core competency. It enables physicians to accurately understand patients’ concerns, symptoms, and needs by demonstrating attentiveness, acknowledging patient input, and responding appropriately to verbal and nonverbal cues (16). Empathy and compassion represent another essential dimension, involving the recognition and validation of patients’ emotions and the provision of empathetic responses, which strengthen trust and rapport (17). Equally important is the ability to communicate medical information clearly and without unnecessary jargon, thereby ensuring that patients comprehend their diagnoses, treatment options, and required follow-up actions (18). Sensitivity to cultural differences is also critical, as it reduces the risk of misunderstandings and promotes effective care for diverse patient populations (19).

In addition, nonverbal communication, such as maintaining appropriate body language, eye contact, and facial expressions, enhances verbal exchanges and conveys empathy and understanding (16). Adaptability and flexibility further strengthen communication by allowing physicians to adjust their approach to the unique needs and contexts of individual patients (20). Beyond clinical encounters, doctors are also expected to enhance patients’ health literacy by empowering them with the knowledge and skills necessary to make informed health decisions (21). Patient education is thus an essential responsibility, reinforced by the provision of constructive feedback and reassurance, which bolsters patients’ confidence in both the healthcare system and their own capacity to manage health concerns (22).

## 4 Developing communication skills and medical education

The refinement of communication skills in medical practice is an ongoing process that builds upon both innate abilities and formal training. This journey begins with foundational instruction in medical school and evolves through clinical practice, residency, and ongoing professional development. While some individuals may have a natural aptitude for effective communication, all medical professionals can benefit from targeted skill enhancement and adaptation to the unique demands of healthcare settings. Deliberate practice, reflection, and structured feedback are essential for improving communication competence, regardless of one’s starting point. This approach acknowledges individual differences in communication abilities while emphasizing the universal importance of ongoing skill development in the medical field.

Therefore, the early acquisition of core communication skills is critical (9, 23), as it prepares future physicians to engage effectively with patients, families, and interdisciplinary healthcare teams throughout their careers. Integrating communication training into the medical curriculum ensures that these skills are taught alongside biomedical knowledge, reinforcing the connection between clinical expertise and patient-centered care. Such programs commonly emphasize active listening, empathy, shared decision-making, and patient education, fostering a holistic approach to health care delivery (16, 20, 24, 25).

Simulation-based learning has emerged as a cornerstone of communication training, enabling students to practice complex conversations and manage challenging scenarios in a controlled environment while receiving structured feedback from instructors and peers (22, 26). Recently, technological innovations such as digital platforms, virtual reality, and interactive e-learning modules have expanded opportunities for skill development. These tools provide flexible and repeatable scenarios and allow analytical tracking of performance, enhancing self-reflection and instructor-guided evaluation (27).

Collectively, these approaches underscore the importance of embedding communication training throughout medical education, ensuring that future physicians are not only clinically competent but also equipped to communicate effectively, foster patient trust and improve overall healthcare outcomes.

## 5 Evaluation of communication skills during medical education

Assessment represents a critical dimension of communication skills education, as it ensures that students not only acquire knowledge but also apply it effectively in clinical practice. Clinical simulations have emerged as one of the most widely used and effective methods for evaluating medical students’ communication skills. These simulations provide structured yet authentic patient interactions in which students can demonstrate their capacity to convey information clearly, respond empathetically and navigate complex clinical scenarios (25, 28, 29). By creating a safe environment for practice, simulations allow students to learn from their mistakes without compromising patient safety and receive immediate feedback from instructors.

In addition to simulation-based assessments, self-and peer assessments are often incorporated into communication training. These approaches encourage students to critically reflect on their communication styles, interpersonal effectiveness, and areas for improvement, fostering a habit of continuous self-evaluation and professional growth (30). Structured assessment tools, such as questionnaires and standardized rating forms, are frequently used to provide objective performance measures. These instruments may be administered in paper form or electronically and are designed to capture both students’ self-perceived competencies and their observed behaviors during interactions with standardized patients or in clinical settings (31).

## 6 Communication skills among medical doctors and students in Greece

The literature on the evaluation of communication skills among medical doctors and students in Greece remains relatively limited, highlighting a critical area for further research and curriculum development. One of the earliest studies, conducted by Liangas and Lionis (32), emphasized that effective communication between practitioners and patients is a defining feature of primary care in Greece. The study underscored the urgent need for more structured vocational training for general practitioners, with particular emphasis on communication competencies, noting that such skills are often underdeveloped in routine practice. Several years later, Tsimtsiou et al. (33) identified prior training in communication skills as a key predictor of positive attitudes toward sharing information with patients, demonstrating the long-term impact of early skill development on later attitudes. Similarly, Oikonomidou et al. (34) reported that although Greek physicians acknowledge the importance of effective communication, they do not consistently employ evidence-based approaches when delivering bad news, indicating gaps between theoretical knowledge and practical application. More recently, Louizou et al. (35) found that most physicians are unfamiliar with the concept of health literacy, which limits their ability to promote it in clinical encounters and hinders patient empowerment.

In recent years, the research focus has increasingly shifted toward medical students, reflecting the importance of developing communication skills early in training. Voultsos et al. (36) investigated empathy levels among Greek medical students, finding that female students scored significantly higher than their male counterparts. Complementing this, Avlogiari et al. (37) demonstrated that intensive experiential training enhances students’ empathy levels, highlighting the value of hands-on learning. Gardikioti et al. (38) observed that students who volunteered during the COVID-19 pandemic recognized the need for additional communication training as a crucial component of their education. Similarly, Savvidou et al. (39) reported that final-year students self-identified the “basic principles of doctor-patient communication” as one of their least developed competencies.

Taken together, these findings highlight the critical need to incorporate structured, evidence-based communication training into Greek medical education, ensuring that future physicians are equipped with patient-centered philosophy of care and are capable of effectively fostering health literacy.

## 7 Integration of communication training in Greek medical school curricula

The Greek medical curriculum spans 6 years, with students primarily engaged in preclinical and theoretical coursework during the first 4 years of the program. Substantial clinical exposure occurs only in the final 2 years, when students interact extensively with patients, making the development of communication skills during this period particularly relevant and reflective of the real-world clinical practice.

However, the findings in Table 1 suggest that formalized communication training remains limited within Greek medical curricula. Only three of the seven operating medical schools offer a dedicated module on communication, and among these, only two are designated as core modules, indicating that most students are not guaranteed structured exposure to these essential skills. Furthermore, the allocated ECTS for these modules is minimal. For example, a three ECTS module represents only 0.83% of a typical 360 ECTS medical program, highlighting that communication constitutes a very small proportion of the overall curriculum. While dedicated modules are scarce, it is plausible that communication competencies are embedded within other mandatory courses, such as “Medical Psychology.” However, without a standardized, explicit focus, the delivery and depth of this training remain inconsistent and are difficult to assess.

**Table 1:** Modules in communication in medical curricula in Greece.

Medical school	Modules title/module type/semester/ECTS
National and Kapodistrian University of Athens	–
Aristotle University of Thessaloniki	Communication with patient/Core/3rd/3
University of Ioannina	Empathy in the doctor-patient relationship/Elective/7th/2
University of Crete	Patient-centered care: concepts and principles/Core/8th/2
University of Patras	–
University of Thessaly	–
Democritus University of Thrace	–

## 8 Discussion

This perspective underscores that Greece lags behind in the formal development of communication skills within medical education, as such skills are not consistently offered as stand-alone courses in medical curricula. However, this is not unique to Greece. Across Europe, communication skills are often embedded within broader training modules, such as palliative care, interdisciplinary teamwork, and patient-centered care strategies (40). For example, preclinical dental education in Europe has faced challenges in ensuring the systematic inclusion of communication training (41). In contrast, in the United States, communication skills are more frequently recognized as a core component of medical education. Best practices, such as “medical improv,” which draws on improvisational theatre to strengthen doctor-patient communication, have gained traction (42). Moreover, these curricula often emphasize the integration of communication training within broader competencies, including evidence-based medicine, thereby reinforcing both the technical and interpersonal dimensions of clinical practice (43).

This limited emphasis is particularly concerning, as communication skills are essential not only for clinical effectiveness but also for supporting patients’ emotional and psychological wellbeing. Moreover, provider self-disclosure and empathetic engagement have been associated with higher patient satisfaction and improved perceptions of care, helping to build rapport and trust (44). The increasing integration of technology, such as Electronic Health Records, further underscores the need for training that combines technical proficiency with patient-centered communication to maintain meaningful interactions (45). Health literacy represents another critical dimension. Currently, its development rests almost exclusively on physicians, with barriers such as time constraints, systemic inefficiencies, and organizational pressures impeding its effective promotion (35, 46). Therefore, integrating health literacy training into both undergraduate and continuing professional education is essential.

In this context, health communication serves as a bridge connecting medical education with broader public health goals, equipping future physicians to deliver patient-centered care while advancing public health outcomes. By strengthening these communication skills within medical education programs, from undergraduate medical curricula through residency training and continuing medical education, the healthcare educational system can better prepare graduates to not only treat individual illness but also to act as agents of public health, fostering health literacy and empowering patients. Nevertheless, while effective communication is a cornerstone of patient care and health literacy, it operates within a complex ecosystem of socio-economic determinants, healthcare policies, and systemic barriers. Therefore, enhancing communication skills should be considered a critical yet complementary strategy alongside broader public health interventions.

To equip future physicians with strong communication skills for both individual patient care and broader public health impact, early exposure to these skills and continuous learning throughout medical education are fundamental. Programs such as “Basics and Practice in Communication Skills” demonstrate that longitudinal training significantly enhances students’ competencies (47). Thus, a systematic integration of communication and health literacy training into Greek medical curricula is urgently needed. Embedding these competencies as core longitudinal components, supported by standardized assessment, simulation-based learning, and faculty development, can ensure that graduates are fully prepared for modern clinical practice. Real-world practice, coupled with regular feedback from peers, supervisors, and patients, can further refine skills such as active listening, empathy, and cultural competence (16, 25), while methods such as small-group teaching can also improve outcomes but require increased faculty resources (39). Implementing these changes presents practical challenges, notably the need for increased faculty resources and dedicated funding to support these initiatives. Potential solutions could involve “train-the-trainer” programs to build faculty capacity efficiently and leverage technology-enhanced learning, such as validated virtual reality simulations, to provide scalable, standardized practice opportunities without overwhelming faculty time. Collectively, these interventions ensure that graduates can contribute effectively to both individual and population health education.

This perspective is limited by its reliance on an analysis of curriculum content and existing literature without incorporating primary data from curriculum developers, faculty, or students. Thus, the findings may not fully capture the implicit or experiential dimensions of communication skills training in Greek medical education. Future research should address these gaps by conducting in-depth qualitative studies in Greek medical schools to examine how communication skills are taught, perceived and experienced in practice. In addition, longitudinal research following medical students from their preclinical years through residency would provide valuable evidence on the development of communication competencies over time and inform evidence-based curriculum reform.

## 9 Conclusion

In conclusion, transforming medical education by integrating communication and health literacy is no longer an enhancement but a fundamental necessity in modern healthcare. The current Greek medical curriculum, with its limited structured training, scarcity of dedicated modules, and minimal credit allocation, is inadequate to equip future physicians with these essential competencies. To address this, medical schools must pivot from viewing these skills as supplemental to embracing them as core and longitudinal components of medical education. This requires a systematic reform that embeds communication training throughout the preclinical and clinical years, supported by faculty development and standardized assessment. By doing so, the educational system can cultivate physicians who are not only expert clinicians but also proficient communicators and advocates of health literacy. Prioritizing these educational interventions will bridge the critical gap between individual patient care and broader public health objectives. Ultimately, fostering these skills is essential for preparing graduates who can build patient trust, enhance treatment adherence, and meet the demands of a modern healthcare system, thereby securing better health outcomes for individuals and populations.

## Data Availability

The original contributions presented in the study are included in the article/supplementary material, further inquiries can be directed to the corresponding author.
